# Alterations in vaginal microbiome in women with short cervix: longitudinal study of microbial diversity and impact of vaginal progesterone treatment

**DOI:** 10.1002/uog.29269

**Published:** 2025-06-26

**Authors:** E. Celik, G. Ozcan, C. Vatansever, E. Paerhati, L. Uygur, C. Unal, S. Guler Cekic, M. A. Ozten, A. Gürsoy, Ö. Keskin, M. Turgal, T. Gursoy, F. Can

**Affiliations:** ^1^ School of Medicine, Department of Obstetrics and Gynecology Koc University Istanbul Türkiye; ^2^ Department of Medicine, Division of Gastroenterology and Hepatology University of Illinois at Chicago Chicago IL USA; ^3^ Koç University İşBank Research Center for İnfectious Diseases (KUISCID) Istanbul Türkiye; ^4^ School of Medicine, Department of Medical Microbiology Koc University Istanbul Türkiye; ^5^ Koc University, College of Engineering Istanbul Türkiye; ^6^ Zeynep Kamil Women and Children Diseases Training and Research Hospital Istanbul Türkiye; ^7^ School of Medicine, Department of Intensive Care Unit and Neonatology Koc University Istanbul Türkiye

**Keywords:** cervical length, pregnancy, preterm birth, progesterone, vaginal microbiome

## Abstract

**Objectives:**

A short cervix is a known risk factor for preterm birth, and imbalances in the vaginal microbiome, such as low relative abundance of *Lactobacillus*, may be associated with an increased risk of preterm birth. The aim of this study was to evaluate differences in the vaginal microbiome between women with a short cervix and those with normal cervical length in the second trimester. Additionally, we aimed to assess longitudinal changes in microbial diversity during pregnancy, as well as the impact of vaginal progesterone treatment on vaginal microenvironment in women with a short cervix.

**Methods:**

This was a prospective, longitudinal study conducted at Koc University Hospital between January 2020 and May 2023, in women with a singleton pregnancy with a short cervical length (≤ 25 mm) in the second trimester (20 + 0 to 24 + 6 weeks' gestation). After diagnosis of short cervix, administration of 200 mg vaginal progesterone daily was initiated. The control group comprised women with a normal cervical length (> 25 mm) in the second trimester, matched for age and body mass index (BMI). Cervicovaginal swabs were collected from the posterior fornix at three gestational‐age ranges: in the first trimester (11 + 0 to 13 + 6 weeks), the second trimester (20 + 0 to 24 + 6 weeks) and the third trimester (28 + 0 to 34 + 6 weeks), and cervical length was measured following sample collection. DNA was extracted and the 16S rRNA bacterial gene was sequenced to analyze and compare the vaginal microbiome between women with a short cervix and controls. We also assessed the microbiome longitudinally in each group, across the first, second and third trimesters. In the short‐cervix group, we also compared the microbiome before initiation of progesterone treatment in the second trimester and 4 weeks after its initiation.

**Results:**

Among 490 pregnant women who underwent first‐trimester screening during the study period and had vaginal swabs collected, short cervical length was detected in 31 at the second‐trimester scan. These women formed the study group. A further 27 women, with a normal cervical length, were matched for BMI and age and assigned to the control group. During the second trimester, women with a short cervix exhibited greater species diversity compared with the control group; this was suggested by the higher Shannon index (0.45 *vs* 0.33; *P* = 0.135), which reflects species richness and evenness, and further demonstrated by the higher Chao index (20.2 *vs* 13.8; *P* = 0.018), which estimates species richness. In the second trimester, *Lactobacillus* was less abundant in women with a short cervix than in the control group, although the difference did not reach significance (86.8% *vs* 95.5%; *P* = 0.091). At the phylum level, in women with a short cervix compared to those with normal cervical length, the relative abundance of Firmicutes, to which the genus *Lactobacillus* belongs, was significantly lower (90.7% *vs* 97.6%; *P* = 0.041), while the relative abundances of both Bacteroidota (1.73% *vs* 0.4%; *P* = 0.004) and Proteobacteria (0.2% *vs* 0.01%; *P* = 0.007) were higher. In the second trimester, the relative abundance of *Lactobacillus gasseri* was significantly lower in women with a short cervix compared to controls (4.7% *vs* 13.8%; *P* = 0.023). In the longitudinal analysis of the vaginal microbiome, there were no significant differences among the trimesters in the control group. In contrast, in those with a short cervix, there was a notable decrease in the amount of *Lactobacillus crispatus*, from 55.0% in the first trimester to 36.1% in the second trimester (*P* = 0.052). In women with a short cervix, there was no significant difference in bacterial diversity after *vs* before progesterone treatment (Chao index, 22.6 *vs* 20.5; *P* = 0.609).

**Conclusion:**

These findings highlight the significant alterations in the vaginal microbiome of pregnant women with a short cervix in comparison to those with normal cervical length, particularly in terms of higher species diversity and distinct community composition. The study also shows that vaginal progesterone treatment in women with a short cervix does not alter the vaginal microbiome, suggesting that it is a safe and effective intervention without disrupting the vaginal microbial balance. Understanding the relationship between cervical length and the vaginal microbiome is essential for developing strategies to reduce the risk of preterm birth in high‐risk populations. © 2025 The Author(s). *Ultrasound in Obstetrics & Gynecology* published by John Wiley & Sons Ltd on behalf of International Society of Ultrasound in Obstetrics and Gynecology.

## INTRODUCTION

Preterm delivery accounts for a significant proportion of perinatal mortality and morbidity worldwide and poses a major healthcare challenge^1^. Preterm birth not only puts infants at risk of immediate health complications but also exacerbates long‐term health challenges and developmental disabilities. In view of such serious complications and the economic burden of preterm birth, there is a pressing need for effective approaches to reduce the rate of preterm birth and associated neonatal morbidity^1^, ^2^.

Multiple mechanisms, including infection or inflammation, uterine overdistention and decidual senescence, have been implicated in the initiation of spontaneous preterm birth^3^. In normal pregnancy, the amniotic cavity is considered ‘sterile’, but microbial invasion of the amniotic cavity, which is subclinical in nature, occurs in approximately one in every four preterm births^4^. Changes in the vaginal microbial ecosystem have been implicated in the development of ascending intrauterine infection^4^, ^5^, ^6^, ^7^, ^8^. The vaginal microbiome plays a crucial role in pregnancy outcome. Studies have shown that the vaginal microbial communities of pregnant women are dominated by *Lactobacillus* species and are characterized by lower species richness and diversity but greater stability than are those of non‐pregnant women^9^. However, during pregnancy, there can be a transition in vaginal *Lactobacillus* species, and a lower relative abundance of *Lactobacillus* species has been observed in the vaginal microbiome of patients who experienced preterm birth in comparison with women who delivered at term^9^.

A short cervix is a well‐established risk factor for preterm birth, and emerging evidence suggests that an imbalance in the vaginal microbiome, characterized by a longitudinal decrease in *Lactobacillus*‐dominated communities during pregnancy, may also be associated with an increase in the risk of preterm birth^10^, ^11^. Preterm birth is a complex process associated with various contributing factors and is a significant concern in obstetrics. One approach to prevent preterm birth is vaginal progesterone treatment^12^. The aim of this study was to evaluate differences in the vaginal microbiome between women with a short cervix and those with normal cervical length in the second trimester (20 + 0 to 24 + 6 weeks), to assess longitudinal changes in microbial diversity during pregnancy and to determine the effect of progesterone treatment on the vaginal microbiome in women with a short cervix.

## METHODS

This longitudinal prospective study was conducted at Koc University Hospital between January 2020 and May 2023. The study was approved by the Koc University Ethics Board (No: 2019. 093. IRB2.030) and registered as a clinical trial (No: NCT04165252).

Women with a singleton pregnancy were invited to participate and assessed for eligibility in the first trimester (11 + 0 to 13 + 6 weeks' gestation) after providing written informed consent to participate. Final recruitment was conducted in the second trimester. Maternal demographic characteristics, including age, height, weight and obstetric history, were recorded. Body mass index (BMI) was calculated using the formula: maternal weight/(height^2^). Pregnancy outcomes were obtained from hospital records. Gestational age was determined based on the last menstrual period and further confirmed through the measurement of fetal crown–rump length during the first‐trimester scan.

The study group included all women with a short cervical length (≤ 25 mm) identified in the second trimester (20 + 0 to 24 + 6 weeks' gestation). Following diagnosis of a short cervix, these women received 200 mg vaginal progesterone daily. The control group included women with normal cervical length (> 25 mm) in the second trimester, matched by age and BMI. Both groups received identical antenatal care. This included follow‐up visits for ultrasound assessment of fetal growth carried out every 4 weeks until 34 weeks' gestation. Exclusion criteria were: major fetal anomaly or chromosomal abnormality, history of organ transplant, chronic steroid use, history of preterm birth, multiple gestation, diabetes mellitus, maternal age < 18 years, cervical cerclage *in situ*, uterine anomaly, myoma uteri, use of antibiotics or antifungals within 2 weeks before sample collection, sexual intercourse within 72 hours before sample collection and ongoing progesterone treatment.

For the purposes of this study, from every participant, cervicovaginal swabs were collected in the first (11 + 0 to 13 + 6 weeks) and second (20 + 0 to 24 + 6 weeks) trimesters and, when possible, in the third trimester (28 + 0 to 34 + 6 weeks). Following sample collection, cervical length measurements were also made. Furthermore, following second‐trimester identification of a short cervix, cervical length in these women was measured 4‐weekly to determine whether steroid administration for fetal lung maturation should be commenced. If, after 26 weeks' gestation, the cervical length was < 10 mm, steroids were administered. The sampling and cervical length measurement were carried out as follows. The patient was asked to empty her bladder and she was placed in the lithotomy position. Cervicovaginal swabs were collected from the posterior fornix using a Remel™ BactiSwab™ (Lenexa, KS, USA). All samples were frozen immediately and stored at − 80°C until DNA extraction. Following sample collection, a sagittal view of the endocervical canal was obtained using a Voluson E8 Expert (GE Healthcare, Zipf, Austria) ultrasound machine equipped with a 4–9‐MHz transvaginal transducer. Cervical length measurements were performed by one of two maternal–fetal medicine specialists (E.C. and M.T.), in accordance with ISUOG guidelines^13^. Each examination lasted approximately 3 min. The shortest of the three measurements between the internal os and the external os was recorded.

### DNA extraction from vaginal swabs and 16S rRNA sequencing

DNA extraction from the samples was conducted using the DNeasy PowerSoil Kit (Qiagen, Hilden, Germany), following the manufacturer's protocols. For library preparation, the QIAseq 16S/ITS Panel Kit (Qiagen) was used to sequence the V1–V9 region of the 16S rRNA bacterial genes, enabling the identification of bacteria in the sample. Sequencing was performed using the MiSeq platform (Illumina, San Diego, CA, USA).

### Bioinformatics

FASTQ files obtained from sequencing were demultiplexed using the GeneGlobe Data Analysis Center (Qiagen). The paired‐end FASTQ files containing V1–V2 region sequences were subsequently used to analyze the microbiome of the samples using Mothur software (v.1.45.3)^14^. To ensure the quality of the data, high‐quality sequences were aligned with the SILVA bacterial reference database (v.138.1)^15^. Chimeric sequences were identified and removed from the dataset using the VSEARCH program, which was integrated into the Mothur software^14^. Taxonomic annotation was then assigned to the sequence using the Wang approach in Mothur software^14^, with the SILVA^15^ database serving as the reference for taxonomic classification. For further analysis of diversity and composition, the sequences were clustered into operational taxonomic units based on a 3% dissimilarity threshold. To determine the classification of all vaginal samples, we applied the community state types (CST) classification proposed by France *et al*.^16^, using the VAginaL community state typE Nearest CentroId clAssifier (VALENCIA), a method based on the nearest centroid approach. CSTs are characterized by microbiome composition. CST I is dominated by *Lactobacillus crispatus*, CST II by *Lactobacillus gasseri*, CST III by *Lactobacillus iners* and CST V by *Lactobacillus jensenii*. CST IV, in contrast, is characterized by a diverse mix of bacteria rather than by *Lactobacillus* species and has a high relative abundance of *Gardnerella vaginalis*.

### Statistical analysis

Categorical variables were summarized as number with percentage. Continuous variables were summarized using mean ± SD, or median and interquartile range if there was evidence of skewness. The vaginal microbiome was analyzed and compared between women with a short cervix and controls with normal cervical length in the second trimester. In each group, we assessed the microbiome longitudinally across the first, second and third trimesters. In the short‐cervix group, we also compared the microbiome before initiation of progesterone treatment in the second trimester with the microbiome 4 weeks after progesterone initiation. Alpha diversity, reflecting within‐sample diversity, was assessed using the Shannon index, which incorporates both species richness (number of taxa) and evenness (relative abundance of taxa), and the Chao index, which estimates species richness. To assess the alpha diversity, the relevant indices were calculated using Mothur software, allowing us to explore diversity within individual samples. Beta diversity, which quantifies differences in community composition between samples, was assessed using methods that capture variability in the identity and abundance of taxa across groups. Beta diversity between each sample was calculated using the Bray–Curtis method to quantify the overall differences in bacterial composition between sample types. The significance of group dissimilarity was assessed using the PERMANOVA test. The demographic data and clinical characteristics were compared using Student's *t*‐test and the chi‐square test. The Wilcoxon signed‐rank test for independent variables was performed using Python 3.7 (Python Software Foundation, Beaverton, OR, USA) to evaluate differences in alpha diversity metrics and microbiome composition. A value of *P* < 0.05 indicated statistical significance. All statistical data were visualized using R 4.3.3 (The University of Auckland, Auckland, New Zealand), ImageGP (State Key Laboratory for Quality Ensurance and Sustainable Use of Dao‐di Herbs, National Resource Center for Chinese Materia Medica, China Academy of Chinese Medical Sciences, Beijing, China) and Prism 8.0.2 (GraphPad, San Diego, CA, USA).

## RESULTS

### Study population

**Figure 1:**
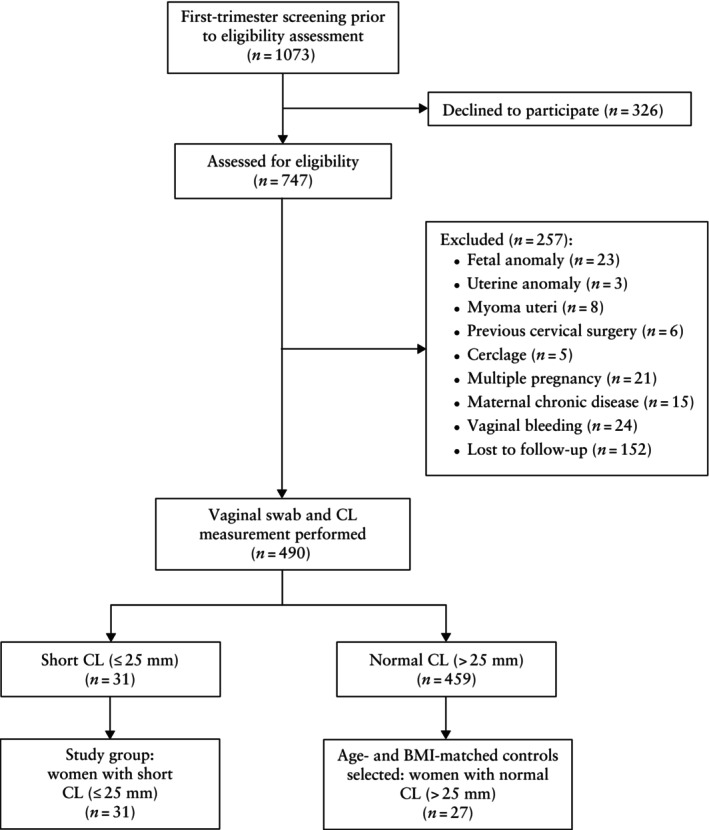
Flowchart summarizing recruitment of pregnant women into study group (short second‐trimester cervical length (CL)) and control group (normal second‐trimester CL). BMI, body mass index.

A flowchart summarizing the study population is presented in Figure 1. Of 1073 women who attended the clinic for a first‐trimester screening test during the study period, 747 women accepted to participate in the study and were assessed for eligibility. In total, 257 women were deemed to be ineligible, and vaginal swabs were collected and cervical lengths were measured for the remaining 490 women. Among these, 31 women were found to have a short cervical length (≤ 25 mm) in the second trimester (20 + 0 to 24 + 6 weeks) and these comprised the study group. A control group of 27 women with normal cervical length was defined, matched to the study group for BMI and age. There were no significant differences in maternal age, BMI or parity between the study and control groups (Table 1). The cervical lengths in the second trimester were significantly different between the groups (*P* < 0.001). Eight women with a short cervix delivered prematurely (< 37 weeks), of whom four delivered before 34 weeks' gestation. All women had samples from cervicovaginal swabs for the first and second trimesters and all women in the control group had third‐trimester samples. Third‐trimester samples could not be collected from 13 women with a short cervix because of preterm delivery or because the patient sought care at an external center.

**Table 1:** Demographic and clinical characteristics of cohort of women with a singleton pregnancy, according to whether cervical length (CL) was short (≤ 25 mm) or normal (> 25 mm) at 20–24 weeks' gestation

Characteristic	Short CL (*n* = 31)	Normal CL (*n* = 27)	*P*
Maternal age (years)	32.4 ± 4.6	30.9 ± 3.8	0.185
Body mass index (kg/m^2^)	23.7 ± 3.9	22.5 ± 3.7	0.428
Parity	1 (0–2)	0 (0–1)	0.898
Cervical length (mm)			
First trimester (11 + 0 to 13 + 6 weeks)	30.5 (28.7–35.2)	36.5 (31.2–40.8)	0.158
Second trimester (20 + 0 to 24 + 6 weeks)	23.3 (21.0–24.2)	32.6 (31.3–34.6)	< 0.001
Third trimester (28 + 0 to 34 + 6 weeks)	20.9 (17.1–23.0)*	32.8 (30.0–37.5)	< 0.001
Gestational age at birth (weeks)	37.3 ± 3.2	39.1 ± 6.7	0.188
Birth weight (g)	3024.4 ± 654.6	3455.8 ± 439.2	0.005
Vaginal delivery	4 (12.9)	8 (29.6)	0.116
1‐min Apgar score	9 (8–10)	9 (8–10)	0.910
Delivery before 37 weeks	8 (25.8)	0 (0)	N/A

Data are presented as mean ± SD, median (interquartile range) or *n* (%), as appropriate.

* Data available in *n* = 18. N/A, not applicable.

### Vaginal microbiome in women with short *vs* normal second‐trimester cervical length

**Figure 2:**
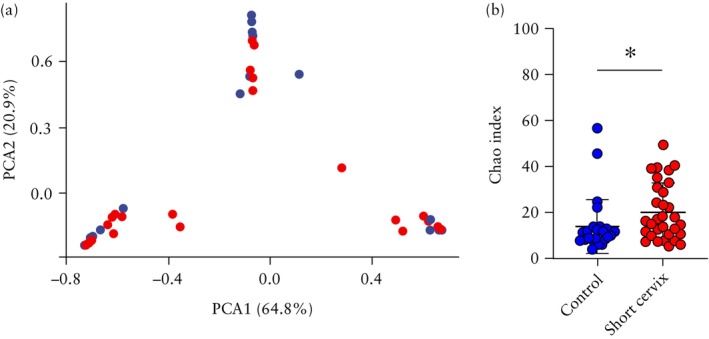
Comparison of alpha and beta diversity of vaginal microbiome between women with normal cervical length (*n* = 27, 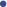) and those with a short cervix (*n* = 31, 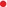) in the second trimester. (a) Beta diversity between each sample was calculated using the Bray–Curtis method to quantify overall differences in bacterial composition between sample types. The significance of group dissimilarity was assessed using the PERMANOVA test. Dispersion *P*‐value = 0.211 (assesses dispersion of a group of samples in ordination space between two groups); adonis *P*‐value = 0.742 (assesses difference in species composition between two groups). Principal components analysis (PCA) illustrates the microbiome composition and assesses clustering of the control and short‐cervix groups based on predefined criteria. Percentages for PCA1 and PCA2 represent contribution of each principal component, indicating how distinctly samples were separated. (b) Comparison of alpha diversity (Chao index). Mean value of each group, with 95% CI, is indicated. *P < 0.05.

The characteristics of bacterial communities in the second trimester of pregnancy were examined by assessing their alpha and beta diversity and examining the differences or similarities in microbial communities between the study and control groups. Beta diversity was not significantly different between the controls and the women with a short cervix (*P* = 0.742) (Figure 2a). Greater species diversity and richness were observed among women with a short cervix compared to controls. This was suggested by the Shannon index (0.45 *vs* 0.33; *P* = 0.135) and was further demonstrated by the Chao index (20.2 *vs* 13.8; *P* = 0.018) (Figure 2b).

**Figure 3:**
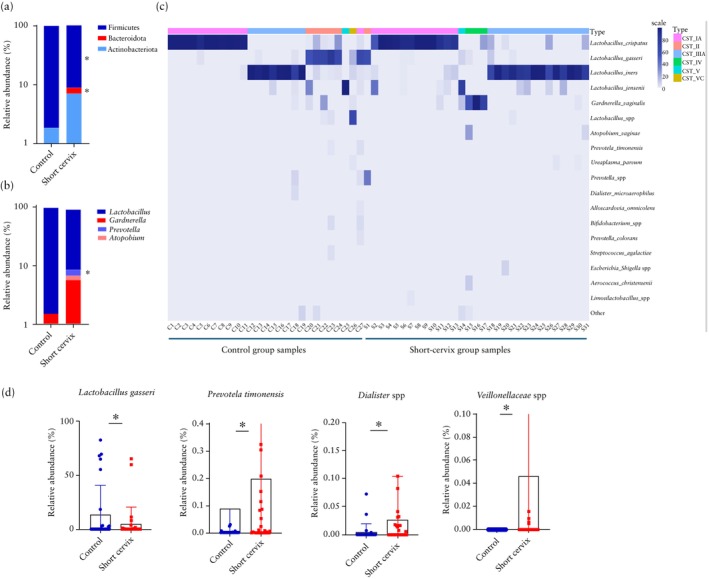
(a,b) Comparison of second‐trimester vaginal microbiome composition in women with a short cervix (*n* = 31) and controls with normal cervical length (*n* = 27). At phylum level (a), Firmicutes and Bacteroidota were significantly different between groups and, at genus level (b), *Prevotella* was significantly different between the two groups (*P < 0.05). Relative abundance is shown on logarithmic scale. Species with < 1% relative abundance are not plotted. (c) Heatmap showing relative abundance of species clustering. Community state types (CST) were classified by VAginaL community state typE Nearest CentroId clAssifier (VALENCIA). CST IA, CST II, CST IIIA, CST IV, CST V and CST VC were defined according to microbiome composition (CST I is dominated by *Lactobacillus crispatus*, CST II by *Lactobacillus gasseri*, CST III by *Lactobacillus iners* and CST V by *Lactobacillus jensenii*; CST IV is characterized by a mix of different bacteria and not by a *Lactobacillus* species). (d) Comparison of relative abundance of species that were significantly different between the two groups (*P < 0.05). Individual samples are plotted for women with short cervix (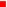) and controls (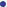); boxes show mean and whiskers show 95% CI.

At the phylum level, in women with a short cervix compared to those with normal cervical length in the second trimester, the relative abundance of Firmicutes, to which the genus *Lactobacillus* belongs, was significantly lower (90.68% *vs* 97.6%; *P* = 0.041), whereas the abundances of both Bacteroidota (1.73% *vs* 0.4%; *P* = 0.004) (Figure 3a) and Proteobacteria (0.2% *vs* 0.01%; *P* = 0.007) were higher. The relative abundance of Actinobacteria was higher in women with a short cervix than in the control group, although the difference was not statistically significant (7.11% *vs* 1.87%; *P* = 0.490) (Figure 3a).

At the genus level, in the second trimester, *Lactobacillus* was less abundant in women with a short cervix than in the control group, although this difference did not reach significance (86.8% *vs* 95.5%; *P* = 0.091) (Figure 3b). The relative abundance of *Gardnerella* (7.8% *vs* 1.5%; *P* = 0.511) and *Atopobium* (1.07% *vs* 0.06% *P* = 0.520) was similar between the groups (Figure 3b), as was that of *Aerococcus* (0.34% *vs* 0.01%; *P* = 0.812). The relative abundance of *Prevotella* was significantly greater in women with a short cervix compared to controls (1.7% *vs* 0.4%; *P* = 0.013) (Figure 3b), as was that of *Escherichia*–*Shigella* (0.267% *vs* 0.0009%; *P* = 0.022).

At the species level, in the second trimester, the relative abundance of *L. gasseri* was significantly lower in women with a short cervix compared to those with normal cervical length (4.7% *vs* 13.8%; *P* = 0.023) (Figure 3d). The relative abundance of *L. crispatus* was also lower in women with a short cervix than in the control group (39.7% *vs* 41.2%; *P* = 0.574), while that of *L. iners* was higher (36.5% *vs* 32.2%; *P* = 0.55), although these differences were not statistically significant (Figure 3c). Furthermore, *G. vaginalis* (7.77% *vs* 1.5%; *P* = 0.520), *Atopobium vaginae* (0.96% *vs* 0.06%; *P* = 0.599) and *Prevotella* spp. (1.34% *vs* 0.31%; *P* = 0.254) were enriched in women with a short cervix compared to the control group, but these differences were not statistically significant. The relative abundances of *Prevotella timonensis* (0.19% *vs* 0.08%: *P* = 0.012), *Dialister* spp. (0.02% *vs* 0.004%; *P* = 0.021) and *Veillonellaceae* spp. (0.05% *vs* 0.0001%; *P* = 0.03) were significantly greater in women with a short cervix compared to controls (Figure 3d).

### Longitudinal analysis of vaginal microbiome

**Figure 4:**
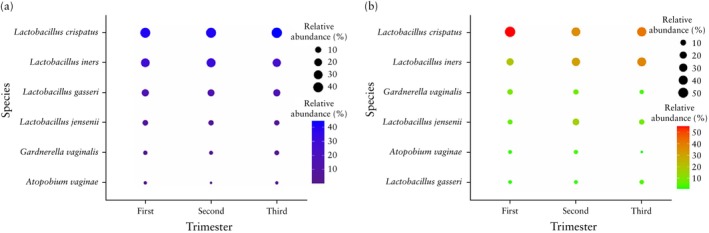
Vaginal microbiome composition trends of 25 women with normal (a) and 10 women with short (b) cervix in first, second and third trimesters. Both scales (i.e. scales of color and of circle size) reflect relative abundance of species in each trimester.

The longitudinal analysis of the vaginal microbiome was performed in 10 women with a short cervix and 25 controls. In pregnant women with normal cervical length in the second trimester, the vaginal microbiome showed very little difference among the trimesters (Figure 4a). *L. crispatus* was dominant in all trimesters (relative abundance in trimesters 1, 2 and 3: 40.6%, 40.8% and 44.4%, respectively) (*P* = 0.081), followed by *L. iners* (28.5%, 31.0% and 25.0%, respectively) (*P* = 0.310), *L. gasseri* (16.1%, 14.7% and 15.8%, respectively) (*P* = 0.962) and *L. jensenii* (7.1%, 5.8% and 5.6%, respectively) (*P* = 0.898). In contrast, in the longitudinal analysis of the vaginal microbiome from women with a short cervix (Figure 4b), there was a notable decrease (although this did not reach statistical significance) in the relative abundance of *L. crispatus* from the first trimester (55.0%) to the second trimester (36.1%) (*P* = 0.052), then a slight increase in the third trimester (to 41.2%) (*P* = 0.133). In contrast, there was an increase from the first to the second trimester in the relative abundances of *L. iners*, *L. jensenii* and *A. vaginae*, although only that of *L. jensenii* reached statistical significance. Specifically, *L. iners* had a relative abundance of 20.1% in the first trimester *vs* 30.9% in the second trimester (*P* = 0.104) and 37.3% in the third trimester (second *vs* third trimester, *P* = 0.455); *L. jensenii* had a relative abundance of 5.9% in the first trimester *vs* 16.5% in the second trimester (*P* = 0.040) and 7.6% in the third trimester (second *vs* third trimester, *P* = 0.126); *A. vaginae* had a relative abundance of 2.09% in the first trimester *vs* 3.3% in the second trimester (*P* = 0.368) and 0.6% in the third trimester (second *vs* third trimester, *P* = 0.621) (Figure 4b).

### Effect of progesterone treatment on vaginal microbiome in women with short cervix

**Figure 5:**
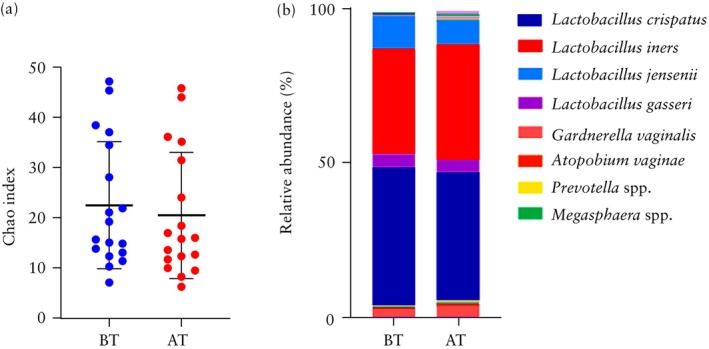
Comparison in 18 women with short cervix before (BT) *vs* after (AT) progesterone treatment. (a) Alpha diversity (Chao index): individual samples are plotted and mean value of each group, with 95% CI, is indicated. (b) Vaginal microbiome composition.

Progesterone treatment was initiated for all 31 women diagnosed with a short cervix in the second trimester. Vaginal samples were successfully collected in the third trimester from 18 of these 31 women, enabling a comparison of samples obtained before *vs* after the initiation of progesterone therapy in these 18 cases. There was no significant difference in bacterial diversity before *vs* after progesterone treatment, as indicated by the species richness and alpha diversity index (mean Chao index, 22.6 *vs* 20.5; *P* = 0.609) (Figure 5a). Furthermore, there were no significant changes after *vs* before initiation of treatment in the relative abundance of bacterial taxa (Figure 5b). The relative abundance of *L. crispatus* was 44.4% before the initiation of progesterone treatment and 41.2% after treatment (*P* = 0.141), while that of *L. iners* was 33.9% before treatment and 37.3% after treatment (*P* = 0.170).

## DISCUSSION

In pregnant women with a short cervix, a notable pattern in the vaginal microbial composition emerged. During the second trimester, the species diversity and richness were greater among women with a short cervix compared to controls, as suggested by the Shannon index (0.45 *vs* 0.33; *P* = 0.135, although the difference was not statistically significant, probably due to limited sample size) and further demonstrated by the Chao index (20.2 *vs* 13.8; *P* = 0.018). In the second trimester, at the genus level, the relative abundance of *Lactobacillus* was lower in women with a short cervix compared to controls; however, the difference was not statistically significant. Imbalances in microbiome composition, particularly a relative decrease in *Lactobacillus* species, have been linked to increased risk of preterm birth^17^, ^18^, ^19^, ^20^, ^21^. Our observation of a tendency towards lower abundance of *L. crispatus*, along with increased abundance of *L. iners* and *L. jensenii*, in women with a short cervix in comparison to the control group suggests a shift toward a microbiome that is less optimal for pregnancy. *L. iners* is associated with an unstable microbial environment, providing less protection than does *L. crispatus*. These differences in women with a short cervix highlight the dynamic nature of the vaginal microbiome and its potential role in influencing cervical health.

The gestational age at delivery was similar between the pregnant women with a short cervix in the second trimester and those with normal cervical length. Yet, several previous studies have demonstrated that a short cervix is associated with an increased risk of preterm delivery^10^, ^22^. A potential explanation for this lack of statistically significant difference in gestational age at delivery in our study may be the relatively small sample size, limiting the study's power to detect such differences and obscuring this relationship^10^, ^22^, ^23^. We were unable to perform a subanalysis using a cervical length cut‐off of 1.5 cm, because the number of patients meeting this criterion was too small (*n* = 3).

Our finding of increased species diversity and decreased relative abundance of *Lactobacillus* in women with a short cervix compared to those with a normal cervical length is consistent with findings from previous research^24^. Callahan *et al*.^20^ found that high relative abundance of *L. crispatus* was associated with a reduced risk of spontaneous preterm birth, whereas *L. iners* did not have the same protective effect in their cohort. Our current study revealed that, in the second trimester, the relative abundance of *Prevotella* at the genus level was significantly higher in women with a short cervix compared to those with normal cervical length; this is consistent with previous research^25^. Although our finding was not statistically significant, the relative abundances of *Gardnerella* and *Atopobium* were also higher in women with a short cervix compared to controls in the present study. These genera are often associated with bacterial vaginosis and a disrupted, dysbiotic vaginal environment^26^. The higher prevalence of these bacteria in women with a short cervix could imply a further departure from the optimal microbiome composition, potentially exacerbating the risks associated with cervical shortening^19^, ^27^. These findings are particularly significant because they suggest a distinct microbial pattern associated with cervical shortening, a condition linked to an increased risk of spontaneous preterm birth. Kindinger *et al*.^27^ reported results concordant with ours, finding that short cervix is associated with low prevalence of *L. crispatus*.

A systematic review and meta‐analysis demonstrated that vaginal progesterone administration reduces the risk of preterm delivery and neonatal morbidity in asymptomatic women with a short cervix^12^. However, some of these women still experience preterm birth despite treatment. Findings of Amorim‐Filho *et al*.^28^ indicated that treatment with an Arabin pessary and natural progesterone does not alter significantly the vaginal microbiome, suggesting that vaginal progesterone does not substantially change microbial composition. We hypothesized that any adverse pregnancy outcomes associated with the presence in the vaginal environment of *L. iners*, which has potential proinflammatory functions, might be mitigated by the anti‐inflammatory properties of progesterone^27^, ^29^ without changing the microbiome composition. In our previous animal experiment, progesterone treatment had no significant effect on the vaginal microbiome composition during pregnancy^30^. Consistent with this previous study, the findings of our current study did not show any effect of vaginal progesterone on the vaginal community structure during pregnancy^27^, implying that progesterone prevents preterm birth through mechanisms unrelated to microbiome modulation.

According to our longitudinal examination of the vaginal microbiome of women with a short cervix, there was a notable reduction in the prevalence of *L. crispatus*, which decreased from 55.0% during the first trimester to 36.1% in the second trimester. Conversely, the relative abundances of *L. iners*, *L. jensenii* and *A. vaginae* increased during this period of pregnancy, although only the increase in *L. jensenii* reached statistical significance. During the second trimester, women whose vaginal microbiota was composed primarily of *L. iners* experienced a significant change in their CST more frequently than did those whose microbiota was dominated by *L. crispatus*. *L. iners* has been recognized as an intermediary phase between lactobacillus dominance and microbiome states associated with CST IV^31^, ^32^. The observed interactions between *L. iners* and the maternal host, which potentially foster a vaginal mucosal environment conducive to colonization by pathogens associated with bacterial vaginosis, may have significant implications for the risk of preterm birth^29^. The propensity of *L. iners* to coexist with these pathogens suggests that *L. iners* has a less protective effect compared with other *Lactobacillus* species, particularly *L. crispatus* ^29^. Therefore, the dominance of *L. iners* in the vaginal microbiome might be an important factor to consider in the screening and prevention of risks associated with preterm birth.

The strength of this study lies in its prospective, longitudinal design, which allowed for data collection at multiple timepoints during pregnancy, providing a comprehensive view of changes in the vaginal microbiome over time. Specifically, collecting cervicovaginal swabs at different gestational stages enabled the assessment of temporal microbiome shifts. Strict inclusion and exclusion criteria minimized confounding factors, enhancing the reliability of the findings.

It is essential to consider these results in the context of the study's limitations, including its specific population and the observational nature of its design. The study might have been limited by the relatively small sample size, potentially affecting the statistical power to detect significant differences. Moreover, conducting the study at a single center may have limited the generalizability of the findings to other populations. Future research should aim to replicate these findings in more diverse populations, to establish a more comprehensive understanding of the impact of vaginal progesterone treatment on the vaginal microbiome.

### Conclusions

Overall, our findings significantly enhance understanding of the vaginal microbiome during pregnancy, particularly regarding complications such as a short cervix. A decrease in *Lactobacillus* species and an increase in pathogenic bacteria can disrupt the vaginal ecosystem and lead to infection, potentially resulting in harm to the developing fetus. It is unclear whether the cervicovaginal microbiome actively causes cervical remodeling or is merely an indication of this. Our study found that vaginal progesterone treatment does not alter significantly the vaginal microbiome in women with a short cervix, suggesting that it is a safe and effective intervention in these women. Understanding the relationship between cervical length and vaginal microbiome composition is crucial for developing strategies to reduce the risk of preterm birth in high‐risk populations.

## Data Availability

The data have been deposited in Zenodo (https://doi.org/10.5281/zenodo.10215255 and https://doi.org/10.5281/zenodo.10455986).
